# Designing High Entropy Bulk Metallic Glass (HE-BMG) by Similar Element Substitution/Addition

**DOI:** 10.3390/ma15051669

**Published:** 2022-02-23

**Authors:** Hongyu Ding, Hengwei Luan, Hengtong Bu, Hongjie Xu, Kefu Yao

**Affiliations:** 1 Marine Equipment and Technology Institute, Jiangsu University of Science and Technology, Zhenjiang 212100, China; 2 School of Materials Science and Engineering, Tsinghua University, Beijing 100084, China; luanhengwei6770@163.com (H.L.); bht20@mails.tsinghua.edu.cn (H.B.); xuhongjie@mail.tsinghua.edu.cn (H.X.)

**Keywords:** high entropy alloy, bulk metallic glass, similar element substitution/addition, glass forming ability, lattice distortion

## Abstract

In this paper, we report that two newly designed high entropy bulk metallic glasses (HE-BMGs), Ti_20_Hf_20_Cu_20_Ni_20_Be_20_ with a critical diameter of 2 mm, and Ti_16.7_Zr_16.7_Nb_16.7_Cu_16.7_Ni_16.7_Be_16.7_ with a critical diameter of 1.5 mm, can be fabricated by copper mold casting method. These newly developed HE-BMGs exhibited a high fracture strength over 2300 MPa. The glass forming ability and atomic size distribution characteristics of the HE-BMGs are discussed in detail. Moreover, a parameter *δ*′ was proposed to evaluate the atomic size distribution characteristics in different HEAs. It showed that this new parameter is closely related to the degree of lattice distortion and phase selection of high-entropy alloys. Adjusting the value of *δ*′ parameter by similar element substitution/addition would be beneficial for designing high entropy bulk metallic glasses.

## 1. Introduction

In the past few decades, bulk metallic glasses (BMGs) [1,2,3,4,5,6,7,8,9] and high entropy alloys (HEAs) [10,11,12,13,14,15,16,17,18] have attracted much attention, owing to their unique structure and properties, such as high strength/hardness, good corrosion/wear resistance, etc. Previously, BMGs and HEAs were developed separately in most cases, following different composition design and fabrication routes. While recent studies show that intersections exist between these two domains, namely some HEAs with meticulously designed composition could be made into BMGs, and hence the high entropy bulk metallic glasses (HE-BMGs) were developed [19,20,21,22,23,24,25,26,27,28,29,30,31,32,33,34,35,36,37,38,39,40,41]. An investigation into HE-BMGs is beneficial for understanding the phase formation rules of HEAs and fundamental issues of BMGs, so it is very important to develop more HE-BMGs.

In our previous work, a Ti_20_Zr_20_Cu_20_Ni_20_Be_20_ HE-BMG with a critical diameter of 3 mm was successfully obtained by copper mold casting method [24]. By introducing Hf as the sixth constituent element, Ti_16.7_Zr_16.7_Hf_16.7_Cu_16.7_Ni_16.7_Be_16.7_ with a critical diameter of 15 mm [25] and a series of Ti_20_Zr_20_Hf_20_(Cu_20__−__x_Ni_x_)Be_20_ HE-BMGs with critical diameters of larger than 12 mm was developed [26,27]. These results indicate that similar element substitution/addition is an effective way for developing new HE-BMGs, just the same as traditional BMGs. Since Hf is an element chemically similar to Zr while Nb and Zr are also very close in the periodic table of elements, it is reasonable to suppose that by substituting Zr with Hf, or by adding Nb in the Ti_20_Zr_20_Cu_20_Ni_20_Be_20_ quinary HEA system, new HE-BMG with good properties can be obtained. Accordingly, two new HEAs, namely Ti_20_Hf_20_Cu_20_Ni_20_Be_20_ and Ti_16.7_Zr_16.7_Nb_16.7_Cu_16.7_Ni_16.7_Be_16.7_, were designed to verify this assumption, and their glass-forming ability, atomic size distribution characteristics, lattice distortion, and phase selection rules of HEAs are discussed in detail.

## 2. Experimental

The master alloy ingots with nominal compositions of Ti_20_Hf_20_Cu_20_Ni_20_Be_20_ and Ti_16.7_Zr_16.7_Nb_16.7_Cu_16.7_Ni_16.7_Be_16.7_ in equal atomic ratio were prepared by arc melting the mixtures of high purity Ti, Hf, Cu, Ni, Zr, Nb plates, and Be granules (purity higher than 99.99 wt.%) within a pure argon gas environment. Cylindrical rods with different diameters were prepared by copper mold injection or suction casting method. Arc melting and casting was conducted on multi-functional high vacuum arc-melting and melt-spinning system, which was produced by SKY Technology Development Corporation, Shenyang, China. The glassy nature of these as-prepared samples was examined by X-ray diffraction (XRD) technique using a Rigaku D/max-RB XRD spectrometry (Rigaku Corporation, Tokyo, Japan) with Cu Kα radiation (λ = 0.15406 nm). Thermal properties of the glassy alloys were examined by a Shimadzu DSC-60 differential scanning calorimeter (Shimadzu Corporation, Kyoto, Japan) instrument under the protection of N_2_ gas (flow rate: 50 mL/min). The applied heating rate was set as 20 K/min. The DSC instrument was calibrated with In and Zn standard specimens. The errors are within ±1 K. Compression tests with specimens of Ø2 × 4 mm and Ø1.5 × 3 mm in size were carried out on WDW-100 testing machine (Shanghai Precision Instrument Co., Ltd, Shanghai, China) under a stain rate of 4 × 10^−4^ s^−1^.

## 3. Results

**Figure 1:**
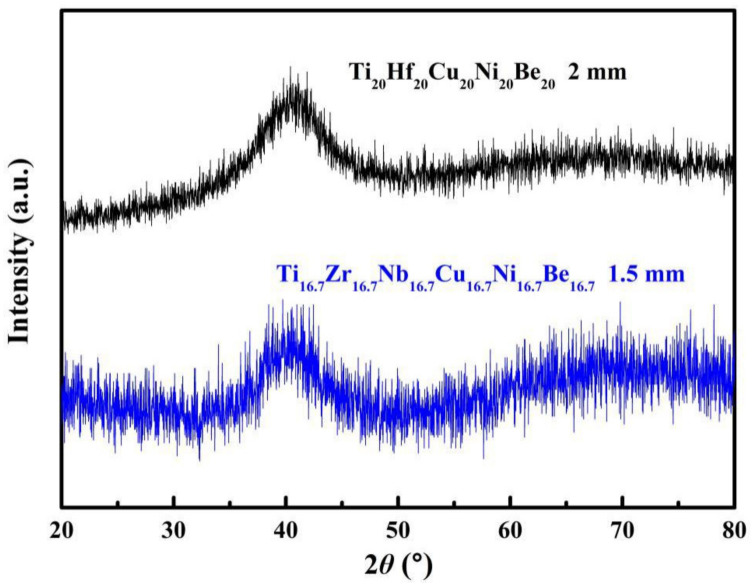
XRD spectra of the Ø2 mm Ti_20_Hf_20_Cu_20_Ni_20_Be_20_ rod sample and Ø1.5 mm Ti_16.7_Zr_16.7_Nb_16.7_Cu_16.7_Ni_16.7_Be_16.7_ rod sample.

Figure 1 shows the XRD spectra of the as-cast Ti_20_Hf_20_Cu_20_Ni_20_Be_20_ and Ti_16.7_Zr_16.7_Nb_16.7_Cu_16.7_Ni_16.7_Be_16.7_ rods with different diameters. No sharp diffraction peak corresponding to the crystalline phase was observed in the Ø2 mm Ti_20_Hf_20_Cu_20_Ni_20_Be_20_ and Ø1.5 mm Ti_16.7_Zr_16.7_Nb_16.7_Cu_16.7_Ni_16.7_Be_16.7_ samples, indicating that they both possess a fully amorphous structure.

**Figure 2:**
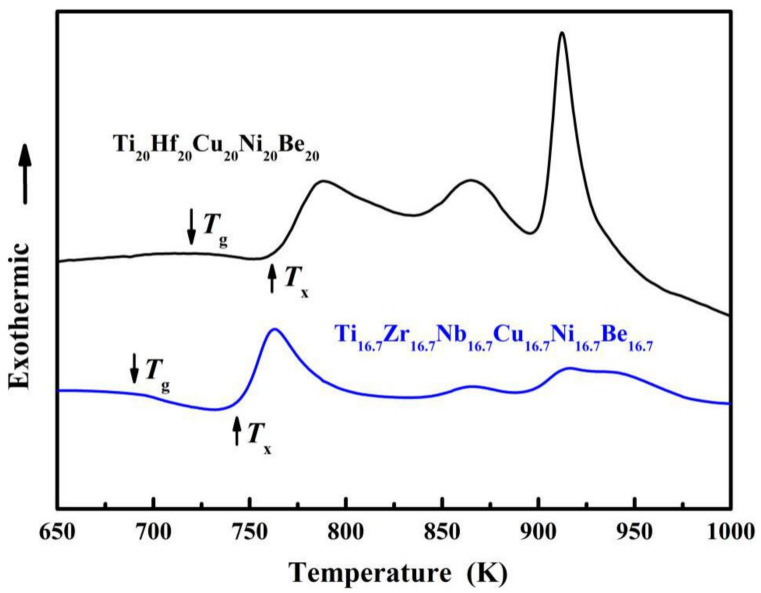
DSC curves of the Ti_20_Hf_20_Cu_20_Ni_20_Be_20_ and Ti_16.7_Zr_16.7_Nb_16.7_Cu_16.7_Ni_16.7_Be_16.7_ HE-BMGs.

The DSC curves of the Ti_20_Hf_20_Cu_20_Ni_20_Be_20_ and Ti_16.7_Zr_16.7_Nb_16.7_Cu_16.7_Ni_16.7_Be_16.7_ samples are shown in Figure 2. The highest test temperature reached 1273 K (1000 °C). However, since the endothermic peak is very high in the high temperature part, glass transition would be very ambiguous in the curve. In order to demonstrate the glass transition phenomenon (which is very important for glasses) clearly, we just cut out temperature less than 1000 K in Figure 2. The glass transition temperature *T*_g_ and initial crystallization temperature *T*_x_ were marked with arrows. *T*_g_, *T*_x_, *T*_m_ (melting temperature) and *T*_l_ (liquidus temperature) were measured as 717 K, 760 K, 1095 K, and 1220 K for the Ti_20_Hf_20_Cu_20_Ni_20_Be_20_ HE-BMG, and 684 K, 739 K, 1066 K, and 1218 K for the Ti_16.7_Zr_16.7_Nb_16.7_Cu_16.7_Ni_16.7_Be_16.7_ HE-BMG, respectively. These data were listed in Table 1. 

**Figure 3:**
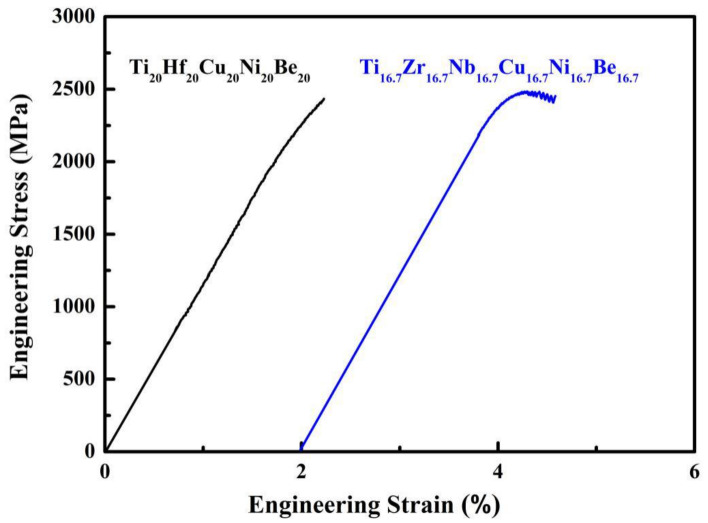
Stress strain curves of the Ti_20_Hf_20_Cu_20_Ni_20_Be_20_ and Ti_16.7_Zr_16.7_Nb_16.7_Cu_16.7_Ni_16.7_Be_16.7_ HE-BMGs.

The stress strain curves of Ø2 × 4 mm Ti_20_Hf_20_Cu_20_Ni_20_Be_20_ and Ø1.5 × 3 mm Ti_16.7_Zr_16.7_Nb_16.7_Cu_16.7_Ni_16.7_Be_16.7_ HE-BMG samples in uniaxial compression test were shown in Figure 3. The fracture strength *σ*_b_ was 2425 MPa for Ti_20_Hf_20_Cu_20_Ni_20_Be_20_ HE-BMG, the yield strength σ_0_._2_, fracture strength *σ*_b_ and plasticity *ε*_p_ were 2330 MPa, 2450 MPa and 0.5% for Ti_16.7_Zr_16.7_Nb_16.7_Cu_16.7_Ni_16.7_Be_16.7_ HE-BMG, respectively, which were also listed in Table 1. The specimens fractured in a shear mode. It is interesting to note that both Ti_20_Hf_20_Cu_20_Ni_20_Be_20_ and Ti_20_Zr_20_Cu_20_Ni_20_Be_20_ quinary HE-BMGs fractured without any plasticity [24], while Ti_16.7_Zr_16.7_Nb_16.7_Cu_16.7_Ni_16.7_Be_16.7_ and Ti_16.7_Zr_16.7_Hf_16.7_Cu_16.7_Ni_16.7_Be_16.7_ senary HE-BMGs exhibited a compressive plasticity of about 0.5%, as well as serration behavior [25]. The reason of this difference remains unclear.

## 4. Discussion

### 4.1. Glass Forming Ability (GFA) of High Entropy Alloys by Element Addition/Substitution

The parameters of supercooled liquid region Δ*T* (= *T*_x_ − *T*_g_), reduced glass transition temperature *T*_rg_ (= *T*_g_/*T*_l_), and γ parameter (= *T*_x_/(*T*_g_ + *T*_l_)) are calculated as 43 K, 0.588, and 0.392 for Ti_20_Hf_20_Cu_20_Ni_20_Be_20_, while 55 K, 0.562, and 0.388 for Ti_16.7_Zr_16.7_Nb_16.7_Cu_16.7_Ni_16.7_Be_16.7_, respectively. Compared with Ti_20_Zr_20_Cu_20_Ni_20_Be_20_ alloy (3 mm), the critical diameter of Ti_20_Hf_20_Cu_20_Ni_20_Be_20_, alloy (2 mm) and Ti_16.7_Zr_16.7_Nb_16.7_Cu_16.7_Ni_16.7_Be_16.7_ (1.5 mm) both decreased. It is noticed that by substitution Zr with Hf, although *T*_rg_ remains the same, Δ*T* and γ decreased; by addition of Nb as the sixth element, both *T*_rg_ and γ decreased, although Δ*T* increased [24]. It implies that the parameter γ is better than *T*_rg_ and Δ*T* in judging the GFA in these high-entropy glassy alloys; meanwhile high entropy is not always beneficial to the GFA of the HEAs. The substitution of element Hf and the addition of Nb brings the liquidus temperature *T*_l_ higher than that of Ti_20_Zr_20_Cu_20_Ni_20_Be_20_ alloy [24]. As a result, the GFA of the HEA was slightly deteriorated. On the other hand, by the addition of Hf as the sixth element, liquidus temperature *T*_l_ was lowered down. Therefore, the GFA of the Ti_16.7_Zr_16.7_Hf_16.7_Cu_16.7_Ni_16.7_Be_16.7_ was greatly improved as compared with Ti_20_Zr_20_Cu_20_Ni_20_Be_20_ alloy [25]. These results indicate that lowering down liquidus temperature would be helpful for enhancing the GFA.

### 4.2. Atomic Radius Characteristics of HE-BMG

The atomic size distribution characteristics of existing HE-BMGs were shown in Table 2. Based on the atomic radius of constituent elements, they were divided into five categories, namely super large atom (*r* > 0.165 nm), large atom (*r* ≈ 0.16 nm), medium atom (*r* ≈ 0.14 nm), small atom (*r* ≈ 0.12 nm), and ultra-small atom (*r* < 0.12 nm). It is noticed that most HE-BMGs were comprised of 3 to 4 categories, except for those containing nonmetal element such as Si, P, B, C, etc [29,33,36]. In high entropy alloys, larger atomic radius difference leads to larger lattice distortion. In case that lattice distortion exceeds some degree, the lattice collapse and amorphous structure formed accordingly. This is in agreement with Zhang’s work [13].

### 4.3. Assessing Degree of Lattice Distortion in High Entropy Alloys by Parameter δ′

The phase formation rule in HEA is of great importance both scientifically and technologically. The formed phase(s) in HEAs (solid solution, intermetallics and amorphous phase) at certain conditions (alloy composition, preparation method, service environment, etc.) remain unknown for most HEAs [10,13,15,18,42]. Many researchers proposed various criteria to solve this problem, such as the *δ-*Δ*H*_mix_ diagram proposed by Zhang et al. [13], VEC criteria proposed by Guo et al. [15], electronegativity mismatch *D*_c_ proposed by Toda-Caraballo et al. [43], etc. Lattice distortion is a crucial factor in HEAs, and it is also very important in determining phase formation. However, the relationship between lattice distortion and phase formation is still not clear. Much research has been devoted to characterizing the degree of lattice distortion, and to further illustrate its correlation with phase formation, such as the *γ* parameter proposed by Wang et al. [44], the *α*_2_ parameter proposed by Wang et al. [45], etc. However, it is far from clearly understanding. Further investigation is still required.

It is noticed from Table 2 that in most HE-BMGs, the atomic radius of the constituent elements atomic sizes distribute in a wide range; while for many solid solution forming HEAs, atomic sizes are more concentrated (especially for CuCoCrNiFe [10] and Cantor alloy [11], they both possess FCC structure, meanwhile atomic size difference of the constituent elements are very small). However, this is a qualitative description, and it is somehow ambiguous. As a result, a quantitative exemplification is needed.

Based on Table 2, here we propose a new parameter *δ*′ to assess the degree of lattice distortion in HEAs. Supposing that a HEA contains N elements, the atomic fractions are c_1_, c_2_ …… c_N_, respectively, and the atomic radii are *r*_1_, *r*_2_ …… *r*_N_ (*r*_1_ < *r*_2_ < …… < *r*_N_), respectively (data from ref. [46]). Then, the average atomic size is defined as $\bar{r}$:

(1) $$\bar{r}=\sum_{1}^{N}c_{i}r_{i}$$

The lattice distortion parameter *δ*′ is defined as

(2) $$\delta^{\prime }=100\;\sum_{1}^{N-1}\frac{c_{i+1}+c_{i}}{2}\;\frac{r_{i+1}-r_{i}}{\bar{r}}$$

In particular, for equal atomic alloy, *δ*′ is given as

(3) $$\delta^{\prime }=\frac{100}{N}\frac{r_{N}-r_{1}}{\bar{r}}$$

**Figure 4:**
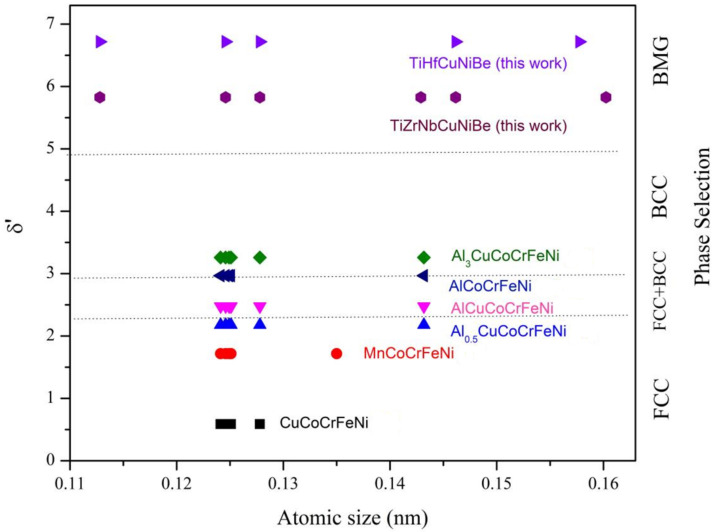
Correlation between atomic size distribution, lattice distortion degree parameter *δ‘* and phase selection in some typical HEAs [10,11,12].

According to Formula (2), lattice distortion parameter *δ*′ for some typical HEAs were calculated and listed in Table 3. For clarity, the relationship between atomic size distribution, lattice distortion parameter *δ*′, and phase selection is demonstrated in Figure 4. It is noticed that *δ‘* is closely related to phase selection in HEAs: when atomic size difference is relatively small, *δ*′ is also small (*δ*′ < 2.2), FCC solid solution would be formed; when the atomic size difference became larger, *δ*′ increased, FCC + BCC solid solution would tend to form as 2.2 < *δ*′ < 2.9; with even larger *δ*′ (2.9 < *δ*′ < 4.9), BCC solid solution would be formed; amorphous phase would be formed as *δ*′ exceeds 4.9.

The parameter *δ*′ can be understood from the point of view of dense atomic packing. Since it is correlated with the degree of lattice distortion, when *δ*′ was small, dense random packing FCC phase formed (its density is about 74% for monolic element); with the increase of lattice distortion, *δ*′ became larger, looser BCC phase (density of about 68% for monolic element) appeared, and its concentration increased accordingly; when lattice distortion became even serious, lattice collapse and amorphous phase would form eventually. Then, adjusting the lattice distortion or the parameter *δ*′ of HEAs, such as by similar element substitution or addition, could be helpful in designing high-entropy metallic glasses.

The new parameter *δ*′ we proposed here is somewhat similar with the *δ* parameter proposed by Zhang et al. [13]; it is also affected by the number, type, and concentration of the elements, while its value is smaller than *δ*, as is demonstrated in Table 3. As compared with *δ*, *δ*′ is more sensitive to addition/substitution of an ultra large/small atom. Taking alloy 11 and 12 in Table 3 for example, it can be seen that by substituting Hf element with much smaller Be element in the Ti-Zr-Hf-Cu-Ni HEA, *δ* increased from 10.324 to 12.514, the growth rate is 21%; while *δ*′ increased from 4.977 to 7.065, the growth rate is 42%, much larger than that of *δ*. It indicates that the new parameter *δ*′ is more sensitive than *δ* in certain circumstance.

Additionally, it is noticed from Formula (3), for equiatomic high entropy alloys, as the number of elements N increased, *δ*′ decreased and lattice distortion is mitigated accordingly. As a result, it is not beneficial for amorphous phase formation, especially for N > 10. This is in consistent with Cantor’s result that an alloy with 16 to 20 elements in equiatomic concentration does not form amorphous phase [11].

## 5. Conclusions

In this paper, two new high entropy bulk metallic glasses (HE-BMGs) have been successfully fabricated using copper mold casting method, namely Ti_20_Hf_20_Cu_20_Ni_20_Be_20_ with a critical diameter of 2 mm and Ti_16.7_Zr_16.7_Nb_16.7_Cu_16.7_Ni_16.7_Be_16.7_ with a critical diameter of 1.5 mm. These two HE-BMGs exhibit high fracture strength over 2300 MPa. The glass forming ability and atomic size distribution characteristics of the HE-BMGs are discussed, and it is found that atomic radius spans over a wide range in HE-BMGs. Moreover, we propose a new parameter *δ*′ to assess the degree of lattice distortion in high entropy alloys (HEAs). It emphasizes the difference between atoms with adjacent atomic size, and it is closely related to phase selection in HEAs. When *δ*′ is relatively small (*δ*′ < 2.2), FCC solid solution formed; when 2.2 < *δ*′ < 2.9, FCC + BCC phases formed; when 2.9 < *δ*′ < 4.9, BCC phase formed; while *δ*′ > 4.9, amorphous phase would be formed. This new parameter *δ*′ is beneficial for understanding lattice distortion and phase selection in HEAs. The present work suggests that through adjusting the parameter *δ*′ by similar element substitution/addition, that is, adjusting the lattice distortion, is an effective way for designing high entropy bulk glassy alloy.

## Figures and Tables

**Table 1:** Thermal and mechanical properties of some HE-BMGs.

Composition	*T*_g_ (K)	*T*_x_ (K)	*T*_m_ (K)	*T*_l_ (K)	*σ*_0_._2_ (MPa)	*σ*_b_ (MPa)	*ε*_p_ (%)	Year
Ti_20_Zr_20_Cu_20_Ni_20_Be_20_	683	729	1076	1161	-	2315	0	2013 [24]
Ti_16.7_Zr_16.7_Hf_16.7_Cu_16.7_Ni_16.7_Be_16.7_	681	751	1019	1100	1943	2064	0.6	2014 [25]
Ti_20_Hf_20_Cu_20_Ni_20_Be_20_	717	760	1095	1220	-	2425	0	This work
Ti_16.7_Zr_16.7_Nb_16.7_Cu_16.7_Ni_16.7_Be_16.7_	684	739	1066	1218	2330	2450	0.5	This work

**Table 2:** Atomic size distribution characteristics of existing HE-BMGs.

Composition	Super Large Atom *r* > 0.165 nm	Large Atom *r* ≈ 0.16 nm	Medium Atom *r* ≈ 0.14 nm	Small Atom *r* ≈ 0.12 nm	Ultra Small Atom *r* < 0.12 nm	Year
Ti_20_Zr_20_Hf_20_Cu_20_Ni_20_		Zr, Hf	Ti	Cu, Ni		2002 [19]
Sr_20_Ca_20_Yb_20_Mg_20_Zn_20_	Sr, Ca, Yb	Mg	Zn			2011 [20,23]
Er_20_Tb_20_Dy_20_Ni_20_Al_20_	Tb, Dy, Er		Al	Ni		2011 [21]
Pd_20_Pt_20_Cu_20_Ni_20_P_20_			Pt, Pd	Cu, Ni	P	2011 [22]
Ti_20_Zr_20_Cu_20_Ni_20_Be_20_		Zr	Ti	Cu, Ni	Be	2013 [24]
Ti_16.7_Zr_16.7_Hf_16.7_Cu_16.7_Ni_16.7_Be_16.7_		Zr, Hf	Ti	Cu, Ni	Be	2014 [25]
Ti_20_Zr_20_Hf_20_(Cu_20−x_Ni_x_)Be_20_		Zr, Hf	Ti	Cu, Ni	Be	2015 [26,27]
Ho_20_Er_20_Co_20_Al_20_Dy_20_	Dy, Ho, Er		Al	Co		2015 [28]
Fe_25_Co_25_Ni_25_(B, Si)_25_				Co, Ni, Fe	Si, B	2015 [29]
Zr_40_Hf_10_Ti_4_Y_1_Al_10_Cu_25_Ni_7_Co_2_Fe_1_	Y	Zr, Hf	Ti, Al	Cu, Co, Ni, Fe		2015 [30]
Er_18_Gd_18_Y_20_Al_24_Co_20_	Y, Gd, Er		Al	Co		2018 [31]
Er_20_Dy_20_Co_20_Al_20_RE_20_ (RE = Gd, Tb, Tm)	Gd/Tb, Dy, Er	Tm	Al	Co		2018 [32]
Fe_25_Co_25_Ni_25_(P_0.4_C_0.2_B_0.2_Si_0.2_)_25_				Co, Ni, Fe	Si, P, B, C	2018 [33]
La_25–35_Ce_25–35_Ni_5–15_Cu_5–15_Al_20_	La, Ce		Al	Cu, Ni		2018 [34]
Fe_25_Co_25_Ni_25_Mo_5_P_10_B_10_			Mo	Co, Ni, Fe	P, B	2019 [35]
(Fe_1/3_Co_1/3_Ni_1/3_)_80_(P_1/2_B_1/2_)_20_				Co, Ni, Fe	P, B	2019 [36]
Zr_35_Hf_17.5_Ti_5.5_Al_12.5_Co_7.5_Ni_12_Cu_10_		Zr, Hf	Ti, Al	Cu, Co, Ni		2019 [37]
Gd_25_Co_25_Al_25_Y_15_RE_10_ (RE = Dy, Ho, Er)	Y, Gd, (Dy, Ho, Er)		Al	Co		2020 [38]
Fe_20–35_Ni_20_Cr_20–30_Mo_5–15_(P_0.6_C_0.2_B_0.2_)_20_			Mo	Cr, Ni, Fe	P, B, C	2020 [39]
(Gd_0.2_Dy_0.2_Er_0.2_Co_0.2_Al_0.2_)_99.5_Si_0.5_	Gd, Dy, Er		Al	Co	Si	2021 [40]
Zr_33_Hf_8_Ti_6_Cu_32_Ni_10_Co_5_Al_6_		Zr, Hf	Ti, Al	Cu, Co, Ni		2021 [41]
Ti_20_Hf_20_Cu_20_Ni_20_Be_20_		Hf	Ti	Cu, Ni	Be	This work
Ti_16.7_Zr_16.7_Nb_16.7_Cu_16.7_Ni_16.7_Be_16.7_		Zr	Ti, Nb	Cu, Ni	Be	This work

**Table 3:** Correlation between atomic size distribution, lattice distortion and phase selection in some typical HEAs.

No.	Composition	*r* > 0.165 nm	*r* ≈ 0.16 nm	*r* ≈ 0.14 nm	*r* ≈ 0.12 nm	*r* < 0.12 nm	$\bar{r}$	*δ* [13]	*δ*′	VEC [15]	Phase
1	CrMnFeCoNi			Mn	Co, Cr, Ni, Fe		1.26744	3.267	1.717	8	FCC [11]
2	CuCoCrNiFe				Cu, Co, Cr, Ni, Fe		1.25304	1.031	0.587	8.8	FCC [10]
3	Al_0.3_CuCoCrFeNi			Al	Cu, Co, Cr, Ni, Fe		1.26315	3.416	2.042	8.472	FCC [10]
4	A_0.5_lCuCoCrFeNi			Al	Cu, Co, Cr, Ni, Fe		1.26928	4.161	2.178	8.273	FCC [10]
5	Al_0.8_CuCoCrFeNi			Al	Cu, Co, Cr, Ni, Fe		1.27768	4.912	2.363	8	FCC + BCC [10]
6	AlCuCoCrFeNi			Al	Cu, Co, Cr, Ni, Fe		1.28281	5.271	2.475	7.833	FCC + BCC [10]
7	Al_2.5_CuCoCrFeNi			Al	Cu, Co, Cr, Ni, Fe		1.31259	6.466	3.106	6.867	FCC + BCC [10]
8	Al_2.8_CuCoCrFeNi			Al	Cu, Co, Cr, Ni, Fe		1.31717	6.554	3.201	6.718	BCC [10]
9	Al_3.0_CuCoCrFeNi			Al	Cu, Co, Cr, Ni, Fe		1.32004	6.598	3.259	6.625	BCC [10]
10	AlCoCrFeNi			Al	Co, Cr, Ni, Fe		1.28378	5.767	2.968	7.2	BCC [12]
11	Ti_20_Zr_20_Hf_20_Cu_20_Ni_20_		Zr, Hf	Ti	Cu, Ni		1.43308	10.324	4.977	-	BMG [19]
12	Ti_20_Zr_20_Cu_20_Ni_20_Be_20_		Zr	Ti	Cu, Ni	Be	1.34318	12.514	7.065	-	BMG [24]
13	Ti_16.7_Zr_16.7_Hf_16.7_Cu_16.7_Ni_16.7_Be_16.7_		Zr, Hf	Ti	Cu, Ni	Be	1.38223	12.773	5.721	-	BMG [25]
14	Ti_20_Hf_20_Cu_20_Ni_20_Be_20_		Hf	Ti	Cu, Ni	Be	1.33818	11.993	6.718	-	BMG (this work)
15	Ti_16.7_Zr_16.7_Nb_16.7_Cu_16.7_Ni_16.7_Be_16.7_		Zr	Ti, Nb	Cu, Ni	Be	1.35495	11.546	5.826	-	BMG (this work)

## Data Availability

The data that support the plots within this paper and other findings of this study are available from the corresponding authors upon reasonable request.
